# Conceptual adequacy of the neuropathic pain symptom inventory in six countries

**DOI:** 10.1186/1477-7525-6-62

**Published:** 2008-08-18

**Authors:** Bruce Crawford, Didier Bouhassira, Audrey Wong, Ellen Dukes

**Affiliations:** 1 Mapi Values, 15 Court Square, Suite 620, Boston, MA, 02108, USA; 2 Hôpital Ambroise Paré, 9, avenue Charles de Gaulle, 92100, Boulogne-Billancourt, France; 3 Pfizer Inc., 235 E 42nd St, New York, NY, 10017, USA

## Abstract

**Background:**

Neuropathic pain results from a nerve lesion or nerve damage. Because it is a subjective experience, patient-reported outcomes may measure both the symptoms and impact on the patient's life. The purpose of this study was to determine whether the Neuropathic Pain Symptom Inventory (NPSI) adequately assesses neuropathic pain symptoms in patients with diabetic peripheral neuropathy, post-herpetic neuralgia, trigeminal neuralgia, and sciatica across multiple cultures.

**Methods:**

From data collected from 132 subjects in 6 countries, qualitative research methods identified their most important symptoms (and verbal descriptions) associated with neuropathic pain. A core set of commonly described symptoms spanning multiple cultures was also described. Moderators using a semi-structured discussion guide conducted focus groups consisting of patients in the U.S., Brazil, Japan, China, Finland, and Spain to elicit concepts that were most important and relevant (concept elicitation phase). Study subjects ranked the importance of each neuropathic pain symptom, completed the NPSI, and commented on its ability to capture key symptoms (face and content validation phase).

**Results:**

Descriptive terms for sensations of neuropathic pain were similar in all countries; burning, electric shocks, and pins and needles were among the most-common sensations. Individuals with neuropathic pain experienced all sensations that were included in the NPSI. They also tended to describe pins and needles and numbness interchangeably, perhaps reflecting the relative number of DPN subjects on study.

**Conclusion:**

Based on data from these focus groups, the NPSI is an acceptable instrument for assessing neuropathic pain.

## Background

Neuropathic pain results from a nerve lesion or nerve damage and may be experienced as burning, electric shock-like, sharp stabbing pains that come and go, deep aches that make sleep or normal activities difficult, or very sensitive skin that reacts to even a slight touch [1,2]. These sensations not only affect the sensory system, but also translate into a wider impact on patients' health related quality of life in terms of alterations in sleep patterns, concentration and mood. Neuropathic pain has been defined by the International Association for the Study of Pain as pain initiated or caused by a primary lesion or dysfunction of the nervous system [3]. Due to the fact that some researchers find this definition overly broad, neuropathic pain has also been characterized as pain caused by lesions of the peripheral or central nervous system (or both) that manifest sensory symptoms or signs [4]. The assessment of neuropathic pain is often complex, given that it is associated with a wide variety of chronic diseases or conditions such as diabetes, carpal or ulnar nerve entrapments, sciatica, spinal cord injury and neuralgia [5].

Neuropathic pain is a subjective experience and the use of patient-reported outcomes (PROs) in measuring symptoms and their manifestation into the patient's life is important. There are several evaluative instruments dealing with neuropathic pain [6-9]. Selecting appropriate measures for the complex assessment of neuropathic pain is challenging. Regulatory agencies have developed guidelines that direct researchers on the development and validation of PRO measures [10,11]. In order for an instrument to be considered well developed, the new guidelines have specified several key points. The development of the instrument must include patient involvement to assist in developing the concepts to be measured or, as the guidelines infer, the question generation process would be incomplete. A wide range of patients should be included in the development of a questionnaire to ensure a representative sample and variations in population characteristics. Following the development of the questions, it is important to review these questions with patients to ensure their clarity and relevance. A questionnaire is not considered valid until the statistical properties have been tested.

The new guidelines direct researchers on the validation steps to ensure the measurement properties are adequate for use in clinical trials. Regulatory agencies want to be sure the questionnaire reliably measures the concepts it was designed to measure. It should be noted, however, that the statistical testing of the questionnaire should guide the development and not dictate which items remain in the questionnaire. Relevance to the patient and clinical importance should always be considered. Most questionnaires were developed solely based on clinical expert opinions regarding which symptoms subjects experience and not the patients' perspective on treatment outcomes – an important scientific standard in questionnaire development [10]. In addition, perceptions and descriptions of neuropathic pain might possibly differ between cultures. Thus, to ensure that the questionnaire is suitable for use in worldwide clinical trials, it should not reflect cultural bias.

This study evaluates the face and content validity of the Neuropathic Pain Symptom Inventory (NPSI) [8]. The NPSI was developed to assess more specifically the different components of neuropathic pain syndromes (i.e. spontaneous ongoing and paroxysmal pain, evoked pain, paresthesia/dysesthesia). This self-questionnaire includes ten items related to different pain descriptors (e.g. burning, squeezing, electric-shock, stabbing, tingling) allowing the assessment of the different dimensions of neuropathic pain and two items on frequency and duration of pain. Each of the items has a recall of the past 24 hours and items are rated on an 11-point numeric rating scale anchored by 0: No (symptom) and 10: Worst (symptom) imaginable. We employed qualitative research methods to identify symptoms deemed most important to the subjects affected by neuropathic pain and the manner in which the subjects describe those symptoms. Because the NPSI may be used to study several forms of neuropathic pain, it is important to establish a core set of neuropathic pain symptoms. Therefore, this assessment focuses on a core set of symptoms commonly described as symptoms in neuropathic pain that also span multiple cultures.

The objective of this study was to determine if the NPSI adequately assesses neuropathic pain symptoms, and is acceptable and relevant to patients with diabetic peripheral neuropathy (DPN), post-herpetic neuralgia (PHN), trigeminal neuralgia (TN), and sciatica across multiple, diverse cultural norms.

## Methods

### Recruitment

Focus groups in six countries (U.S. [English], Brazil [Portuguese], Japan [Japanese], China [Mandarin], Finland [Finnish], Spain [Spanish]) were designed to elicit concepts that were most important and relevant to patients with neuropathic pain. Subjects were recruited through pain specialists via recruitment agencies. The recruitment agencies initiated contact with pain specialists who invited subjects to participate in the study. Subjects received an informational letter outlining the purpose of the study and the extent of their involvement, and physicians obtained informed consent prior to study. Both subjects and their physicians were required to complete a case report form (CRF) that included clinician and subject contact information and ensured the eligibility of the subject through a list of inclusion and exclusion criteria. Subjects were informed that the focus group session would last approximately two hours. The CRFs were reviewed for completeness and patient eligibility prior to beginning the focus group sessions.

Six to ten subjects were recruited for each focus group. An attempt was made to recruit subjects of differing age, gender, and ethnicity (the latter only in the U.S.). Subjects with mild to severe neuropathic pain were included to capture the full spectrum of patient pain.

### Inclusion/exclusion criteria

Study inclusion criteria included: 18 years of age or older; diagnosed with DPN, PHN, TN, or sciatica; able to discern his/her neuropathic pain from any concomitant pain (e.g., joint pain) as determined by their physician; and ability to participate in a two-hour focus group discussion. In addition, subjects met at least three of the following inclusion criteria (abstracted from the ID Pain [12]) to verify the presence of neuropathic pain: described his/her pain as feeling like pins and needles; described his/her pain as feeling like hot/burning; described his/her pain as feeling numb; has described his/her pain as feeling like electrical shocks; and/or reported that his/her pain is worsened by the touch of clothing or bed sheets. Exclusion criteria included: serious mental health or cognition condition(s), including cognitive impairment, severe mental retardation, schizophrenia, and/or physician-assessed clinical depression.

Prior to the initiation of the focus groups, subjects completed forms for informed consent and background demographics, as well as pre-focus group questionnaires. These questionnaires asked subjects to list five terms that describe their nerve pain in conjunction with the five most-bothersome symptoms (i.e., "*People feel pain in many ways and people might describe pain using many different terms. We are interested in how you would describe your nerve pain. Please list below five words that you would use to describe your nerve pain*;" and "*Please list below the three most bothersome sensations you feel related to your nerve pain*."). Collecting this information spontaneously prior to discussing the topic with other subjects via questionnaire avoids the potential introduction of error through "yeah-saying" in the focus groups.

### Concept elicitation and content validation

Trained moderators conducted the focus group sessions using a semi-structured discussion guide. Prior to the start of the focus group, the moderators explained the purpose of the study, reassured the subjects of the confidentiality of their responses, encouraged the subjects to take their time with their responses, and allowed all subjects an opportunity to share their views with the group. The moderator informed the participants that the focus group sessions would be audio- and/or video-recorded as stated in the consent form that each participant had signed prior to the focus group. The focus group guide consisted of: 1) a concept elicitation phase, and 2) face and content validation phase. During the concept elicitation phase, subjects received open-ended questions about their neuropathic pain experiences, focusing on symptoms they experienced due to their neuropathic pain. Subjects identified and described such sensations in detail. Initially, subjects responded spontaneously to these questions. If sensations previously described in the questionnaire were not mentioned spontaneously, the moderator probed the subjects to determine the accurateness of the sensations. These questions were asked prior to the content validation phase of the interview to ensure that the subjects were not unduly biased by the sensations covered in the NPSI. This allowed for a pure assessment of symptoms prior to the face and content validation of the questionnaire and a more guided assessment of symptoms during the second phase of the focus group.

During the concept elicitation phase, the importance of each neuropathic pain symptom was ranked by patients detailing the "most bothersome" sensation they experience. During the face and content validation phase of the focus groups, the subjects completed the NPSI and commented on the extent to which the questionnaire captured key symptoms associated with neuropathic pain. The purpose of this phase of the focus groups was to ensure: 1) the relevance of the concepts covered by the questionnaire, 2) the questionnaire's comprehensiveness and ease of understanding, and 3) the applicability/acceptability of the items.

### Transcription/translation

Transcriptions were produced from the audiotapes of the sessions, and verbatim subject comments were analyzed. Recordings in Japanese, Spanish, Portuguese, and Chinese were transcribed into the respective native language prior to English translation. The English transcripts of the other countries' focus group data were then analyzed. The Finnish tapes were transcribed into Finnish and then analyzed in the native language. Subject quotes were grouped together by symptom and compared to the symptoms included in the NPSI.

Coding schemes were developed to translate descriptions of patient characteristics into thematic trends for data analysis. The thematic coding scheme underwent iterations as the research team coded the preliminary data. Initial coded material was aggregated into broader core categories and analyzed using grounded theory methods [13]. For the concept elicitation sections of the focus groups, each subject comment was assigned a "classification" and "domain" and incorporated into a domain mapping grid. The classifications and domains identified, along with examples of subject quotes, were used as a basis for determining whether all relevant symptoms were included in the NPSI.

## Results and discussion

One hundred and thirty-two subjects from six countries were interviewed (Table 1), Background demographics, including age and gender are summarized in Table 2. The type of neuropathic pain and clinician-rated severity of pain are included in Table 3.

**Table 1:** Focus Group Populations

**Country**	**Number of focus groups**	**Total number of subjects**
United States	6	50
Brazil	1 (plus 10 individual in-depth interviews)^a^	16
China	2	18
Finland	2	17
Spain	2	16
Japan	2	13

^a ^Conducted in place of a focus group due to scheduling conflicts.

**Table 2:** Focus Group Demographics

**Demographic Information**	**U.S.** ** (N = 50)**	**Brazil** ** (N = 16)**	**China** ** (N = 18)**	**Finland** ** (N = 17)**	**Spain** ** (N = 16)**	**Japan** ** (N = 13)**
	n (%)	n (%)	n (%)	n (%)	n (%)	n (%)
**Gender**						
- Female	24 (48)	9 (56)	8 (44)	11 (65)	13 (81)	5 (38)
- Male	26 (52)	6 (38)	9 (50)	6 (35)	3 (19)	8 (62)
- Missing Data	0 (0)	1 (6)	1 (6)	0 (0)	0 (0)	0 (0)
**Age**						
- Range	19–81 years	50–76 years	28–61 years	43–90 years	23–78 years	54–80 years
- Mean	52 years	62 years	47 years	70 years	66 years	66 years
- Median	51 years	61 years	66 years	61 years	72 years	64 years
Education*						
**- **Less than high school	4 (8)	9 (56)	9 (50)	15 (88)	10 (63)	3 (23)
**- **High school diploma/Some college	28 (56)	7 (44)	3 (17)	--	4 (25)	5 (38)
**- **College or university degree (2 or 4 year)	16 (32)	--	6 (33)	--	2 (12)	4 (31)
Marital Status**						
**- **Married	31 (62)	10 (63)	18 (100)	10 (59)	9 (56)	12 (92)
**- **Not married	19 (38)	6 (37)	--	7 (41)	7 (44)	--

* Note: Two patients from the U.S. did not respond; two patients from Finland did not respond; one patient from Japan did not respond.

**Note: One patient from Japan did not respond.

**Table 3:** Focus Group Health Information

**Health ** **Information**	**U.S.** ** (N = 50)**	**Brazil** ** (N = 16)**	**China** ** (N = 18)**	**Finland** ** (N = 17)**	**Spain** ** (N = 16)**	**Japan** ** (N = 13)**
	n (%)	n (%)	n (%)	n (%)	n (%)	n (%)
**Type of Neuropathic Pain**						
- Diabetic Peripheral Neuropathy	18 (36)	14 (88)	5 (28)	0 (0)	1 (6)	5 (38)
- Post-Herpetic Neuralgia	10 (20)	1 (6)	6 (33)	5 (29)	10 (63)	8 (62)
- Trigeminal Neuralgia	8 (16)	1 (6)	7 (39)	12 (71)	4 (25)	0 (0)
- Sciatica	14 (28)	0 (0)	0 (0)	0 (0)	0 (0)	0 (0)
- Missing data	0 (0)	0 (0)	0 (0)	0 (0)	1 (6)	0 (0)
**Clinician-rated pain level**						
- Mild	15 (30)	3 (19)	1 (6)	0 (0)	0 (0)	3 (23)
- Moderate	26 (52)	12 (75)	13 (72)	1 (6)	9 (56)	6 (46)
- Severe	9 (18)	1 (6)	4 (22)	16 (94)	7 (44)	4 (31)

In the U.S., the majority of the subjects (72%) were Caucasian. The remaining participants were African American (8%), Hispanic/Latino (11%), and from other ethnic groups (3%). As illustrated in Table 2, there was some variability by country in both educational level and marital status. Ethnicity was not collected in the other countries due to the ethnic homogeneity for each country. It should be noted that with the exception of the U.S., focus groups were conducted in or around major cities – Sao Paulo, Beijing and Shanghai, Seinajoki (smaller city in western Finland), Madrid and Tokyo.

### Pre-focus group findings

Table 4 summarizes the spontaneous, independent report of symptoms by subjects on the pre-focus group questionnaire, as described in Methods. The most frequently listed words to describe neuropathic pain were "burning," "electric shock," "numbness," and "tingling"; however, not all of the subjects listed sensations.

**Table 4:** Sensations of Neuropathic Pain Included in the NPSI Compared to Sensations Reported on the Pre-Focus Group Questionnaire

**Neuropathic Pain** **Sensations of ** ** Included in the NPSI**	**Sensations of Neuropathic Pain Reported on Pre-Focus Group** ** Questionnaire**					
	**U.S.** ** (n = 50)**	**Brazil** ** (n = 16)**	**China** ** (n = 18)**	**Finland** ** (n = 17)**	**Spain** ** (n = 16)**	**Japan** ** (n = 13)**
Burning	12	5	7	1	5	1
Squeezing	--	--	--	3	--	--
Pressure	1	--	1	3	1	2
Electric Shocks	5	3	5	7	4	5
Stabbing	5	--	7	--	5	1
Pins and Needles	5	--	1	5	--	6
Tingling	6	5	11	2	--	--
**Non-NPSI Sensations**						
Numbness	9	2	7	2	2	3
Prickling	2	--	--	--	--	2
Itchiness	7	--	--	--	8	4
Sharp	9	--	--	--	--	--
Shooting	5	--	--	3	--	--
Throbbing	2	--	--	--	--	--
Stinging	4	1	--	--	--	--
Piercing	--	1	--	--	--	--
Cramps	--	1	--	--	1	--
Cutting	--	--	3	5	--	--
Hot	--	--	1	1	--	--
Pulsating	--	--	--	1	--	--
Drilling	--	--	--	1	--	--

"Squeezing" and "pressure" were the least likely sensations on the NPSI to be elicited spontaneously on the pre-focus group questionnaire. "Pressure" was reported in every country except Brazil and "squeezing" was only mentioned in Finland.

All sensations covered in the NPSI were mentioned spontaneously as being most bothersome on the pre-focus group questionnaire except for squeezing. The most frequent notations of bothersome were burning, tingling, and electric shocks.

### Focus group findings

#### Phase 1

During the focus groups, the most common spontaneous descriptions were burning, electric shocks, numbness, and pins and needles. Subjects often used terms interchangeably; for example, in the U.S., "tingling" and "numbness" were described as "pins and needles."

In Brazil, all symptoms on the NPSI were spontaneously mentioned in the focus group except "squeezing" and "tingling." After probing, subjects also reported experiencing "squeezing." "Tingling" was the only sensation not mentioned by the subjects in Brazil. "Cramps" were described as "similar to twinging" and "coming after the burning pain." After a discussion with a professional translator, it was discovered that "twinging" might be the English translation of the Brazilian word for "tingling." One patient described "twinging" as "stabbing by needles."

In China, subjects also used the terms "heart stabbing," "needle through heart," "tremble," and "bursting" to describe their pain. Interviewers in China noted that these terms should not be interpreted literally. "Bursting" implies a "sudden, strong, and unbearable" feeling of pain. The two terms referring to the heart do not mean that the heart is in pain. When speaking about pain, the Chinese are more likely to relate extreme pain with the heart because they believe the heart is the most critical and sensitive part of the body.

In Spain, the two sensations of "pins and needles" and "stabbing" were combined into one term as "stabbing pins on fire" (n = 8). One subject defined it as if "hundreds, thousands of pins on fire (are) stuck into my body."

Table 5 summarizes the pain sensations experienced by the focus group members that they spontaneously described. The symptoms of the NPSI were consistently reported within the focus groups with the exception of "squeezing." Although "squeezing" was reported in the U.S., Finland and Japan, few subjects stated this as a spontaneous expression of their pain. "Squeezing" was only spontaneously mentioned by one subject and four subjects mentioned "squeezing" while describing other neuropathic pain sensations.

**Table 5:** Sensations Reported in the Focus Groups

**Sensations of** **Neuropathic Pain** ** Included in the NPSI**	**Sensations of Neuropathic Pain Spontaneously Mentioned by Subjects** ** During the Focus Groups**					
	**U.S.** ** (n = 50)**	**Brazil** ** (n = 16)**	**China** ** (n = 18)**	**Finland** ** (n = 17)**	**Spain** ** (n = 16)**	**Japan** ** (n = 13)**
Burning	14	10	2	2	11	--
Squeezing	1	--	--	5	--	1
Pressure	6	3	--	5	5	2
Electric Shocks	10	3	2	10	15	6
Stabbing	6	2	--	1	8	3
Pins and Needles	8	7	6	1	5	4
Tingling	6	2	2	3	1	2

All of the sensations of neuropathic pain included in the NPSI (e.g., burning, squeezing, pressure, electric shocks, stabbing, pins and needles, and tingling) were spontaneously mentioned by subjects during the focus groups. Of the sensations included in the NPSI, "burning," "pins and needles," and "electric shocks" were most frequently mentioned by subjects in the focus groups. Subjects in China did not spontaneously mention three of the seven items (e.g., squeezing, pressure, and stabbing).

In addition to symptoms included on the NPSI, subjects also frequently mentioned "numbness" and "sharp" as sensations they experienced, although "sharp" was only mentioned in the U.S.

Patients in each country consistently described their pain with a single statement. Subjects in the U.S. used "burning," "electric shocks," and "sharp" while those in Spain used "electric shocks" or "sharp" only. Finnish and Japanese subjects also described their pain as "electric shocks," In addition, Japanese subjects used the term, "pins and needles."

The two most bothersome sensations in the U.S. were burning and electric shocks while the two most bothersome sensations in Brazil were cramps and pins and needles. The most bothersome sensations for Spanish subjects were either electric shocks or "stabbing pins on fire." Interestingly, subjects in China defined their worst pain by the emotions they felt or their inability to sleep in addition to the type and duration of the pain episode.

### Review of the Neuropathic Pain Symptom Inventory questionnaire

The majority of the subjects did not raise any concerns with the NPSI: only three subjects mentioned that the recall period was too short, one subject felt that the questionnaire was confusing and another thought it did not capture all of their symptoms. Subjects responded positively when asked if the questionnaire was easy to understand, though one person reported that they did not know what was meant by "squeezing" pain. Next, subjects were asked which words (or kanji characters) they thought were above a sixth-grade reading level. The majority of subjects in other countries stated no concerns; however, words that some U.S. subjects thought were above a six-grade reading level included imaginable, neuropathic, provoke, severity, spontaneous, stimulation, and sensation. Although not thought to be above a six-grade reading level, Japanese subjects suggested that "pressure" was a concept that may be difficult to understand. However, no changes to the NPSI were consistently suggested by focus group subjects.

In China, four subjects felt that the questionnaire did not adequately reflect Chinese and/or Asian culture, and they suggested using a simplified NPSI. Because pain is not judged on a numerical scale, patients did not define their pain in such detail. Instead, subjects in China typically used descriptive terms ("mild," "moderate," or "severe") rather than numbers to quantify pain. However, five individuals felt that the NPSI was an acceptable tool, even if it incorporated a scale to measure pain.

## Conclusion

The focus groups and interviews consisted of 2 phases: 1) concept elicitation, and 2) face and content validation. The information gathered from the focus groups in other countries (e.g., Japan, Brazil, China, Finland, and Spain) was consistent with that from group in the U.S. Descriptive terms for sensations of neuropathic pain were similar in all countries studied. Burning, electric shocks, and pins and needles were among the most-common sensations. Based on feedback from focus group subjects during the concept elicitation phase, all sensations included in the NPSI are indeed experienced by people with neuropathic pain. During the focus groups or individual interviews, subjects used the terms burning, electric shocks, and pins and needles.

Numbness was also consistently mentioned. Although "numbness" is not a true pain descriptor but is related to non-painful paresthesia/dysesthesia, the occurrence of numbness as a frequently reported sensation reflects the number of DPN subjects in the focus groups, as this sensation is typically experienced in DPN. Subjects also used the words numbness and pins and needles interchangeably to describing pain symptoms. Because pins and needles are already included in the NPSI, adding numbness should be considered when a DPN-specific questionnaire is required. Because numbness would not be a component of the validated scoring algorithm, this issue would be considered separately. Similarly, "itchiness" is not a true pain descriptor. In the validation of the NPSI [8], "itchiness" was found to be an unreliable item and therefore was removed. This study, unfortunately, was not designed to evaluate the "global" reliability of responses and therefore, we cannot recommend its inclusion at this time. The descriptor of "squeezing" was not consistently reported across cultures; however, "pressure" was reported more consistently. These two descriptors have been found to belong to the same pain dimension [8] – spontaneous ongoing pain, with similar factor loadings (0.88 and 0.87, respectively). It is therefore thought that these two descriptors will complementarily assess the spontaneous ongoing pain symptoms.

This study was not able to evaluate the differing etiology of pain in the analysis due to the separation of the participant's personal health information from the focus group transcripts. It is likely that subjects across different etiologies describe their pain slightly differently. It would have also been interesting to investigate the terminology utilized by subjects across cultures with the same etiology. As the objective of this study was to evaluate the adequacy of the NPSI for use in different neuropathic pain etiologies in different countries, the results support the broad objective. This is the first study to the knowledge of the authors to confirm such a "universality" of core neuropathic pain descriptors across etiologies and cultures. This study suggests that the small impact of culture on neuropathic pain expression may be related to its specific pathophysiologic mechanism; confirming the notion that neuropathic pain is a specific category of chronic pain that deserves special attention.

In conclusion, the information collected during the focus groups and their analyses demonstrate that the NPSI is an acceptable instrument for assessing neuropathic pain worldwide. Country-specific terms might further enhance its applicability.

## Abbreviations

CRF: Case Report Form; DPN: Diabetic Peripheral Neuropathy; NPSI: Neuropathic Pain Symptom Inventory; PHN: Post-herpetic Neuralgia; PROs: Patient-reported Outcomes; TN: Trigeminal Neuralgia.

## Competing interests

BC and AW are employees of Mapi Values, an outcomes research consulting firm. ED is an employee of Pfizer Inc. DB has received funding for research and speaking engagements from numerous pharmaceutical companies. There are no other competing interests.

## Authors' contributions

BC and ED were responsible for the design and execution of this study. AW was the primary analyst. DB assisted in the interpretation of the results. All co-authors assisted in drafting the manuscript.
